# Outbreaks among Wild Birds and Domestic Poultry Caused by Reassorted Influenza A(H5N8) Clade 2.3.4.4 Viruses, Germany, 2016

**DOI:** 10.3201/eid2304.161949

**Published:** 2017-04

**Authors:** Anne Pohlmann, Elke Starick, Timm Harder, Christian Grund, Dirk Höper, Anja Globig, Christoph Staubach, Klaas Dietze, Günter Strebelow, Reiner G. Ulrich, Jan Schinköthe, Jens P. Teifke, Franz J. Conraths, Thomas C. Mettenleiter, Martin Beer

**Affiliations:** Friedrich-Loeffler-Institut, Greifswald-Insel Riems, Germany

**Keywords:** avian influenza virus, highly pathogenic avian influenza viruses, HPAIV, H5N8, reassortant, viruses, influenza, Germany, wild birds, poultry, clade 2.3.4.4, zoonoses

## Abstract

In November 2016, an influenza A(H5N8) outbreak caused deaths of wild birds and domestic poultry in Germany. Clade 2.3.4.4 virus was closely related to viruses detected at the Russia–Mongolia border in 2016 but had new polymerase acidic and nucleoprotein segments. These new strains may be more efficiently transmitted to and shed by birds.

During 2014–2015, after massive outbreaks of highly pathogenic avian influenza (HPAI) on the Korean Peninsula, subtype H5N8 viruses (group A clade 2.3.4.4) caused outbreaks among wild birds and domestic poultry in central Asia, Russia, and central Europe (*1*,*2*). Strains of this clade, and novel reassortants thereof, were transferred to North America (*3*). Transcontinental spread of these strains and an earlier HPAI virus (HPAIV) of the goose/Guangdong lineage of subtype H5N1 has been linked to dissemination by migratory wild birds (*4*). We describe a novel reassortant of HPAIV A(H5N8) within group B clade 2.3.4.4, which causes lethal infections in hundreds of wild birds and domestic poultry in Germany and elsewhere in Europe.

## The Study

**Figure 1:**
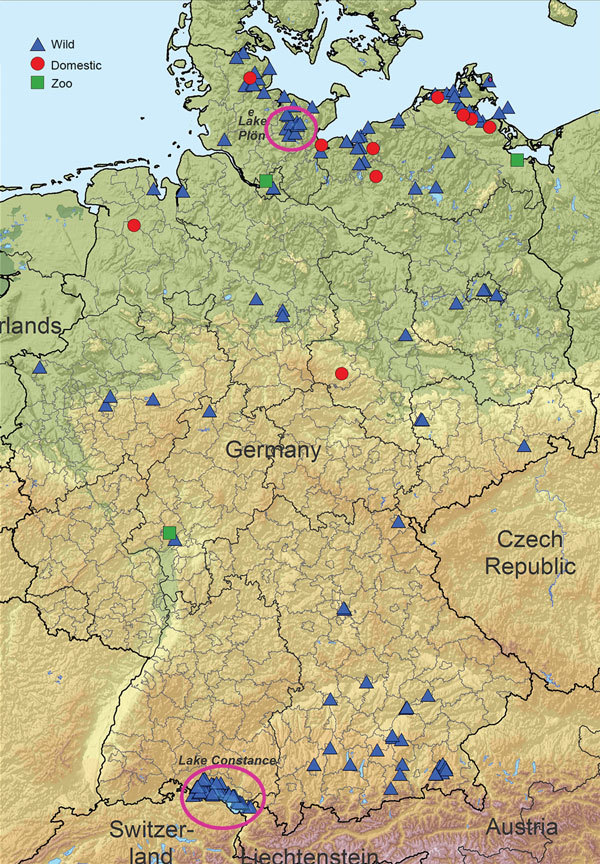
Highly pathogenic avian influenza A(H5N8) cases in wild birds and outbreaks in poultry holdings (10 backyard holdings, 4 zoos or pet farms, and a few commercial operations) in Germany, November 2016. Circles indicate original locations of outbreaks and isolates.

In late May 2016, a group B clade 2.3.4.4 H5N8 virus was detected in dead and hunted wild birds at Lake Uvs-Nuur, at the Russia–Mongolia border (*5*). On November 7, 2016, many dead tufted ducks (*Aythya fuligula*) were found at Lake Plön in Schleswig-Holstein, northern Germany, and at Lake Constance in Baden-Württemberg, southern Germany (Figure 1); most were positive for H5N8. The epidemic among wild birds continued and spread toward the center of the country (Figure 1). As of December 2016, several backyard holdings, 4 zoos, and a few large commercial operations were also affected. Direct or indirect contact with wild birds was the most likely route of virus introduction into the backyard holdings and zoos. Despite the generally high standards of commercial operations, possible biosecurity gaps were identified.

Most affected birds were found dead or exhibited severe clinical signs such as apathy or sudden deaths (in some parts of the affected chicken breeder farms, up to 90% died before culling). Macroscopic changes commonly observed in tufted ducks and poultry included severe diffuse hepatic necrosis, multifocal petechiae, and variably hyperemic and edematous lungs. Light microscopy confirmed influenza A virus nucleoprotein (NP) antigen and variably distinct necrotizing lesions in liver, heart, lungs, brain, pancreas, spleen, and thymus (Technical Appendix Figure 1). Some chickens also displayed severe diffuse catarrhal enterocolitis; influenza A virus NP antigen was present in the intestinal epithelium.

The spectrum of affected species of wild birds is broad and includes mainly diving ducks but also swans, grebes, gulls, buzzards, crows, and a white-tailed eagle (Table 1). As of November 30, 2016, ≈400 infected wild birds were detected in 13 federal states of Germany. H5N8 infections were also reported from Austria, Croatia, Denmark, Finland, France, Hungary, Poland, Romania, Sweden, Switzerland, and the Netherlands, indicating that the same subtype caused the recent outbreaks throughout Europe.

**Table 1:** Species of birds affected by highly pathogenic avian influenza virus A(H5N8), Germany, 2016

Group	Common name (taxonomic name)
Diving ducks	Tufted duck (*Aythya fuligula*)
	Common pochard (*Aythya ferina*)
	Common goldeneye (*Bucephala clangula*)
	Red-crested pochard (*Netta Rufina*)
	Greater scaup (*Aythya marila*)
	Common eider (*Somateria mollissima*)
	Common scoter (*Melanitta nigra*)
Dabbling ducks	Mallard (*Anas platyrhynchos*)
	Northern pintail (*Anas acuta*)
Diving birds	Great crested grebe (*Podiceps cristatus*)
	Little grebe (*Tachybaptus ruficollis*)
Mergansers	Common merganser (*Mergus merganserI*)
Geese	Greylag goose (*Anser anser*)
	Bean goose (*Anser fabalisI)*
	Canada goose (*Branta Canadensis*)
	White-fronted goose (*Anser albifrons*)
	Pink-footed goose (*Anser brachyrhynchus*)
Swans	Mute swan (*Cygnus olorI*)
	Black swan (*Cygnus atratus*)
	Whooper swan (*Cygnus cygnus*)
Gulls	Black-headed gull (*Chroicocephalus ridibundus*)
	European herring gull (*Larus argentatus*)
	Great black-backed gull (*Larus marinus*)
	Mew gull (*Larus canus*)
Rails	Common coot (*Fulica atra*)
Herons	Gray heron (*Ardea cinerea*)
Birds of prey	Common buzzard (*Buteo buteo*)
	Rough-legged buzzard (*Buteo lagopus*)
	White-tailed eagle (*Haliaeetus albicilla*)
Cormorants	Great cormorant (*Phalacrocorax carbo*)
Crows	Carrion crow (*Corvus corone*)
Domestic birds	Domestic duck (*Anas platyrhynchos domesticus*)
	Domestic chicken (*Gallus gallus domesticus*)
	Turkey (*Meleagris gallopavo*)
Zoo birds	Emu (*Dromaius novaehollandiae*)
	Great white pelican (*Pelecanus onocrotalus*)

The high pathogenicity for gallinaceous poultry was confirmed; intravenous pathogenicity index for 1 isolate (A/tufted duck/Germany-SH/AR8444/2016) was 2.93, comparable to the 2.81 index for H5N8 circulating in 2014 (A/turkey/Germany-MV/AR2472/2014). However, deaths of wild birds of a variety of species, in particular diving ducks, and extended pathologic changes in dead wild birds suggested a marked shift of pathogenicity from the viruses in Germany in 2014 (*1*).

For genetic characterization, we analyzed virus sequences from several swab samples and the first 2 virus isolates from dead tufted ducks from Lake Plön and Lake Constance and compared them with sequences from H5N8-positive turkeys and chickens. We found few genetic differences between the analyzed strains from northern and southern Germany and only slight differences between sequences generated from wild bird or poultry samples. This finding clearly contrasts with those of the H5N1 clade 2.2 outbreaks in Germany in 2006, which showed similar distribution (*6*) but 2 distinct H5N1 northern and southern subclusters and a difference in timing during the first transcontinental wave; virus was found in Europe in October 2005 but not in Germany until early 2006.

**Figure 2:**
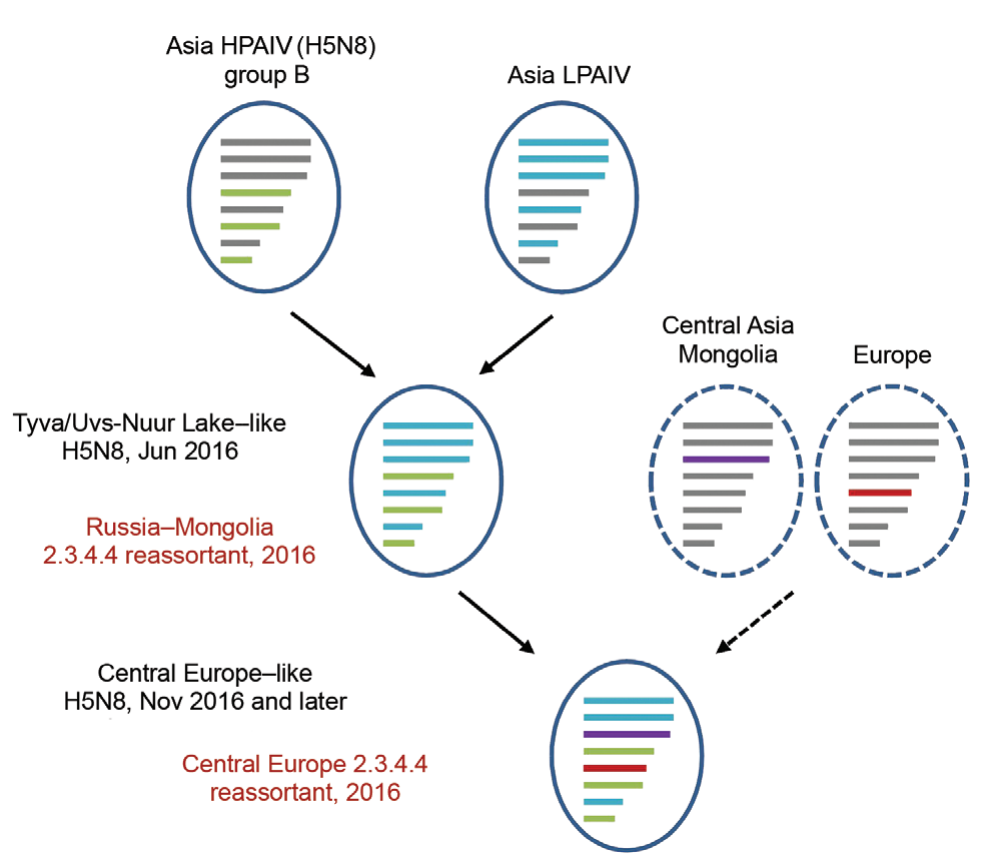
Proposed reassortment events leading to the novel central Europe HPAIV A(H5N8) clade 2.3.4.4 virus. The Russia–Mongolia reassortant clade 2.3.4.4 H5N8 virus acquired 2 new segments (polymerase acidic protein and nucleoprotein), leading to the novel central Europe clade 2.3.4.4 H5N8 in 2016. Similar segment origins are marked by similar colors. Dashed lines indicate putative precursors. HPAIV, highly pathogenic avian influenza virus; LPAIV, low pathogenicity avian influenza virus.

All genome segments of the novel H5N8 clade 2.3.4.4 group B strains from Germany in 2016 differed significantly from the H5N8 clade 2.3.4.4 group A strains detected in 2014–2015 in Germany and other European countries (*1*). This finding is in accord with results of studies suggesting that there was no continued circulation of group A–like viruses among wild birds in the Netherlands from mid-November 2014 to January 2016 (*7*). Database searches identified H5N8 clade 2.3.4.4 viruses (first detected in wild birds at the Russia–Mongolia border at the end of May 2016) as closest relatives. Extended searches confirmed that the isolates from Germany in 2016 described in this study are novel reassortants, which can be clearly distinguished from the isolates from Russia–Mongolia in 2016 (e.g., A/great crested grebe/Uvs-Nuur Lake/341/2016) by 2 segments (Table 2; Technical Appendix Figure 2). Six segments (polymerase basic [PB] 2, PB1, hemagglutinin, neuraminidase, matrix protein, nonstructural protein [NS]) were highly similar to those of clade 2.3.4.4 viruses from Russia in 2016 (99% for each surface protein; Table 2). The NS1 protein of the new 2016 isolate from Germany is truncated (217 aa) compared with the Russia–Mongolia viruses (230 aa), a also truncation found in other influenza virus strains. However, the nuclear export protein is not affected. Of note, the Russia–Mongolia virus proved to be a novel reassortant from earlier H5N8 clade 2.3.4.4 viruses within group B (Table 2; Figure 2) (*5*). These new H5N8 viruses are central Asia reassortants, which originated from strains circulating in eastern Asia. Genes of 3 segments (hemagglutinin, neuraminidase, and NS protein) cluster with segments of H5N8 clade 2.3.4.4 group B viruses identified in eastern China; the other 5 genes (PB1, PB2, polymerase acidic [PA], nucleoprotein [NP], and matrix protein) cluster with avian influenza viruses of low pathogenicity, which were identified in Mongolia, China, and Vietnam (*5*).

**Table 2:** Genetic composition of influenza virus A/tufted duck/SH-Germany/R8444/2016 isolated in Germany, 2016*†

Genome segment, virus strain	Identity, %	Group
PB2		
A/wild duck/Poland/82A/2016 (H5N8)	99	Russia–Mongolia 2.3.4.4 2016 reassortant
A/duck/Mongolia/30/2015 (H3N8)	97	
A/duck/Mongolia/118/2015 (H4N6)	97	
A/great crested grebe/Uvs-Nuur Lake/341/2016 (H5N8)	97	
PB1		
A/wild duck/Poland/82A/2016 (H5N8)	99	Russia–Mongolia 2.3.4.4 2016 reassortant
A/great crested grebe/Uvs-Nuur Lake/341/2016 (H5N8)	99	
A/duck/Mongolia/179/2015 (H3N8)	97	
A/duck/Mongolia/518/2015 (H10N3)	97	
PA		
A/wild duck/Poland/82A/2016 (H5N8)	100	Central Europe 2.3.4.4 2016 reassortant
A/mallard/Republic of Georgia/13/2011 (H6N2)	97	
A/duck/Mongolia/179/2015 (H3N8)	97	
A/duck/Hokkaido/W9/2015 (H1N1)	97	
A/greylag goose/Iceland/0921/2011 (H6N8)	97	
HA		
A/wild duck/Poland/82A/2016 (H5N8)	99	Russia–Mongolia 2.3.4.4 2016 reassortant
A/tufted duck/Denmark/17740–1/2016 (H5N8)	99	
A/mute swan/Croatia/70/2016 (H5N8)	99	
A/great crested grebe/Uvs-Nuur Lake/341/2016 (H5N8)	99	
A/duck/Eastern China/S1109/2014 (H5N8)	98	
NP		
A/wild duck/Poland/82A/2016	99	Central Europe 2.3.4.4 2016 reassortant
A/mallard/Republic of Georgia/13/2011 (H6N2)	98	
A/chicken/Netherlands/16007311–037041/2016 (H7N9)	97	
A/chicken/France/150169a/2015 (H5N1)	97	
A/greylag goose/Iceland/0921/2011 (H6N8)	97	
NA		
A/wild duck/Poland/82A/2016 (H5N8)	99	Russia–Mongolia 2.3.4.4 2016 reassortant
A/great crested grebe/Uvs-Nuur Lake/341/2016 (H5N8)	99	
A/mute swan/Croatia/70/2016 (H5N8)	99	
A/duck/Eastern China/S1109/2014 (H5N8)	98	
MP		
A/wild duck/Poland/82A/2016 (H5N8)	99	Russia–Mongolia 2.3.4.4 2016 reassortant
A/great crested grebe/Uvs-Nuur Lake/341/2016 (H5N8)	99	
A/mute swan/Croatia/70/2016 (H5N8)	99	
A/duck/Mongolia/179/2015 (H3N8)	98	
NS		
A/wild duck/Poland/82A/2016 (H5N8)	99	Russia–Mongolia 2.3.4.4 2016 reassortant
A/great crested grebe/Uvs-Nuur Lake/341/2016 (H5N8)	99	
A/mute swan/Croatia/70/2016 (H5N8)	99	
A/duck/Eastern China/S1109/2014 (H5N8)	99	

* Sequences were compared with entries in the GISAID (http://platform.gisaid.org) database. Details and acknowledgments in online Technical Appendix (https://wwwnc.cdc.gov/EID/article/23/4/16-1949-Techapp1.pdf). HA, hemagglutinin; MP, matrix protein; NA, neuraminidase; NS, nonstructural protein; NP, nucleoprotein; PA, polymerase acidic protein; PB, polymerase basic protein.

The viruses from Germany (2016) further evolved from the strains from Russia and harbor 2 new segments (PA, NP). The virus sequences are essentially identical to sequences from an H5N8 virus isolate found November 2016 in a dead wild duck in Poland near the Germany border (Table 2) but differ significantly from sequences of other currently circulating isolates (Technical Appendix Figure 2). The new PA sequences cluster with sequences of viruses detected in eastern and central Asia. The NP segment sequences are similar to those of viruses frequently found in central and northwestern Europe. The PA and NP segments in question occurred concurrently in different avian influenza viruses before; namely, in H6N8 strains from Iceland in 2011 (e.g., A/greylag goose/Iceland/0921/2011) and in an H6N2 isolate from Georgia in 2011 (A/mallard/Republic of Georgia/13/2011) (Table 2; Technical Appendix Figure 2). It is reasonable to suggest that the novel reassortant strain from Germany was generated by >1 reassortment event that occurred during June–November 2016 between central Asia (Mongolia) and central Europe (Poland/Germany).

## Conclusions

A new reassortant influenza A(H5N8) virus is responsible for the recent HPAIV outbreak in Germany. The observed differences in pathogenicity for a broad spectrum of waterfowl compared with that of H5N8 viruses from 2014–2015 correlate with a new genome composition of these viruses. The novel NP and PA segments in the 2016 H5N8 viruses from Germany are candidates for future studies of the molecular basis of the biological differences. These new strains may be more efficiently transmitted by and shed to other wild and domestic birds, a hypothesis in line with the large number of cases among wild birds in November 2016. There is yet no indication that mammals (including humans) are infected by these novel strains. Future studies in mammalian models (e.g., ferrets, mice) will provide experimental data on the virulence for mammals.

Technical Appendix Additional methods and results for study of reassorted influenza (H5N8) virus causing massive outbreaks among wild birds and domestic poultry, Germany, 2016.
