# Supplementary material for: Outbreaks among Wild Birds and Domestic Poultry Caused by Reassorted Influenza A(H5N8) Clade 2.3.4.4 Viruses, Germany, 2016
Source: Emerg Infect Dis. 2017 Apr;23(4):633–6. doi: 10.3201/eid2304.161949 (PMC5367393; doi:10.3201/eid2304.161949)
Supplement: Technical Appendix — Additional methods and results for study of reassorted influenza (H5N8) virus causing massive outbreaks among wild birds and domestic poultry, Germany, 2016. [file 16-1949-Techapp-s1.pdf]

Article DOI: http://dx.doi.org/10.3201/eid2304.161949

Outbreaks among Wild Birds and Domestic Poultry Caused by Reassorted Influenza A(H5N8) Clade 2.3.4.4 Viruses, Germany, 2016

Technical Appendix

Methods

RNA extracted from swabs or isolates was tested positive for influenza A(H5N8) virus by using reverse transcription quantitative PCR, conventional reverse transcription PCR, and Sanger sequencing as previously described (*1*,*2*). Isolates were obtained after the first passage in 9 day old embryonated eggs from specific pathogen free (SPF) chicken flocks (VALO biomedical) using standard procedures. Undirected shot-gun Next generation sequencing was done as described (*3*) using IonTorrent barcodes on an IonTorrent PGM (Thermo Scientific, Waltham, MA USA). Raw sequence data and phylogenetic analysis were done using Newbler in Genome Sequencer software (v. 3.0; Roche, Mannheim Germany) and Geneious software suite (v. 9.1.6; Biomatters, Auckland, New Zealand). Phylogenetic analyses were done with RAxML (*4*).

Sequences were deposited into the GISAID EpiFlu database under Accession EPI_ISL_237732, EPI_ISL_237733, EPI_ISL_237792, EPI_ISL_237944, EPI_ISL_237945, EPI_ISL_237958, EPI_ISL_238037, EPI_ISL_238038, EPI_ISL_238039, EPI_ISL_238040.

References

1. Starick E, Lange E, Fereidouni S, Bunzenthal C, Höveler R, Kuczka A, et al. Reassorted pandemic (H1N1) 2009 influenza A virus discovered from pigs in Germany. J Gen Virol. 2011;92:1184–8. PubMed http://dx.doi.org/10.1099/vir.0.028662-0

2. Harder T, Maurer-Stroh S, Pohlmann A, Starick E, Höreth-Böntgen D, Albrecht K, et al. Influenza A(H5N8) Virus Similar to Strain in Korea Causing Highly Pathogenic Avian Influenza in Germany. Emerg Infect Dis. 2015;21:860–3. PubMed http://dx.doi.org/10.3201/eid2105.141897

3. Juozapaitis M, Aguiar Moreira É, Mena I, Giese S, Riegger D, Pohlmann A, et al. An infectious bat-derived chimeric influenza virus harbouring the entry machinery of an influenza A virus. Nat Commun. 2014;5:4448. PubMed http://dx.doi.org/10.1038/ncomms5448

4. Stamatakis A. RAxML version 8: a tool for phylogenetic analysis and post-analysis of large phylogenies. Bioinformatics. 2014;30:1312–3. PubMed http://dx.doi.org/10.1093/bioinformatics/btu033

Technical Appendix Table. Acknowledgment of authors and originating and submitting laboratories of the sequences from the GISAID EpiFlu Database

Isolate ID	Isolate name	Originating Lab	Submitting Lab	Authors
EPI_ISL_224580	A/great crested grebe/Uvs-Nuur Lake/341/2016	Research Institute of Experimental and Clinical Medicine	Research Institute of Experimental and Clinical Medicine	Kirill, Sharshov; Olga, Kurskaya; Ivan, Sobolev; Alexander, Alekseev; Tatyana, Alikina; Marsel, Kabilov; Alexander, Shestopalov
EPI_ISL_230820	A/great crested grebe/Tyva/34/2016	State Research Center of Virology and Biotechnology Vector	WHO National Influenza Centre Russian Federation	Fadeev, Artem; Komissarov, Andrey; Egorova, Anna; Sintsova, Ksenia; Musaeva, Tamila; Susloparov, Ivan; Marchenko, Vasiliy; Ryzhikov, Aleksandr
EPI_ISL_231684	A/wild duck/Tyva/35/2016	State Research Center of Virology and Biotechnology Vector	WHO National Influenza Centre Russian Federation	Fadeev,Artem; Komissarov,Andrey; Egorova,Anna; Sintsova,Ksenia; Musaeva,Tamila; Susloparov,Ivan; Marchenko,Vasiliy; Ryzhikov,Aleksandr
EPI_ISL_231685	A/black-headed gull/Tyva/41/2016	State Research Center of Virology and Biotechnology Vector	WHO National Influenza Centre Russian Federation	Fadeev,Artem; Komissarov,Andrey; Egorova,Anna; Sintsova,Ksenia; Musaeva,Tamila; Susloparov, Ivan; Marchenko,Vasiliy; Ryzhikov,Aleksandr
EPI_ISL_234057	A/gray heron /Uvs-Nuur Lake/20/2016	Research Institute of Experimental and Clinical Medicine	Research Institute of Experimental and Clinical Medicine	Kirill, Sharshov; Olga, Kurskaya; Ivan, Sobolev; Alexander, Alekseev; Alexander, Shestopalov
EPI_ISL_234058	A/common tern /Uvs-Nuur Lake/26/2016	Research Institute of Experimental and Clinical Medicine	Research Institute of Experimental and Clinical Medicine	Kirill, Sharshov; Olga, Kurskaya; Ivan, Sobolev; Alexander, Alekseev; Tatyana, Alikina; Marsel, Kabilov; Alexander, Shestopalov
EPI_ISL_237921	A/wild duck/Poland/82A/2016		National Veterinary Research Institut Poland, PIWet-PIB	Świętoń, E.; Śmietanka, K.
EPI_ISL_237922	A/tufted duck/Denmark/17740–1/2016	Technical University of Denmark	Technical University of Denmark	Hjulsager, CK; Krog, JS; Kvisgaard, LK; Larsen, LE
EPI_ISL_238196	A/mute swan/Croatia/70/2016		Croatian Veterinary Institute	Savić, Vladimir
EPI_ISL_238197	A/mute swan/Croatia/78/2016		Croatian Veterinary Institute	Savić, Vladimir

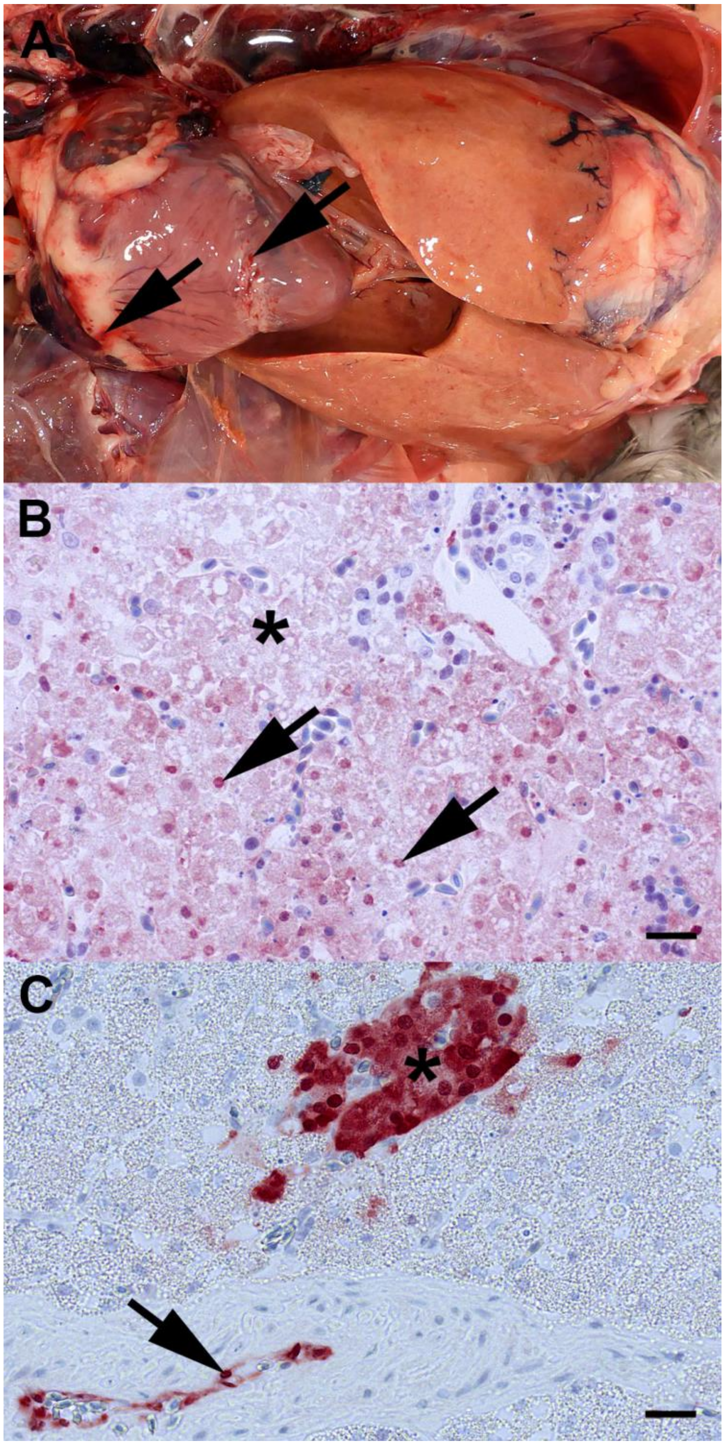

Technical Appendix Figure 1. Pathological findings in Tufted ducks (Aythya fuligula). A) Coelomic cavity. Diffusely ochre-colored, slightly swollen and friable liver (severe acute diffuse hepatic necrosis) and mild acute epicardial petechiae (arrows) represent typical gross changes. B) Liver. Avian influenza virus (AIV)-nucleoprotein- antigen is mainly present within hepatocellular nuclei (arrows) and cytoplasm at

the border of coalescing necrotic lesions (star), characterized by disintegrating cytoplasmic membranes, vesiculated cytoplasm and pyknotic, karyorrhectic and lytic nuclei. C) Pancreas. AIV-nucleoprotein-antigen is also present in epithelial nuclei and cytoplasm within discrete foci of pancreatic acinar cells (star) as well as vascular endothelia (arrow). B, C) Immunohistochemistry; polyclonal rabbit anti- influenza A FPV/Rostock/34-virus-nucleoprotein antiserum; avidin-biotin-complex method; 3-amino-9-ethyl-carbazol chromogen (red); hematoxylin counterstain (blue); scale bars indicate20 μm.

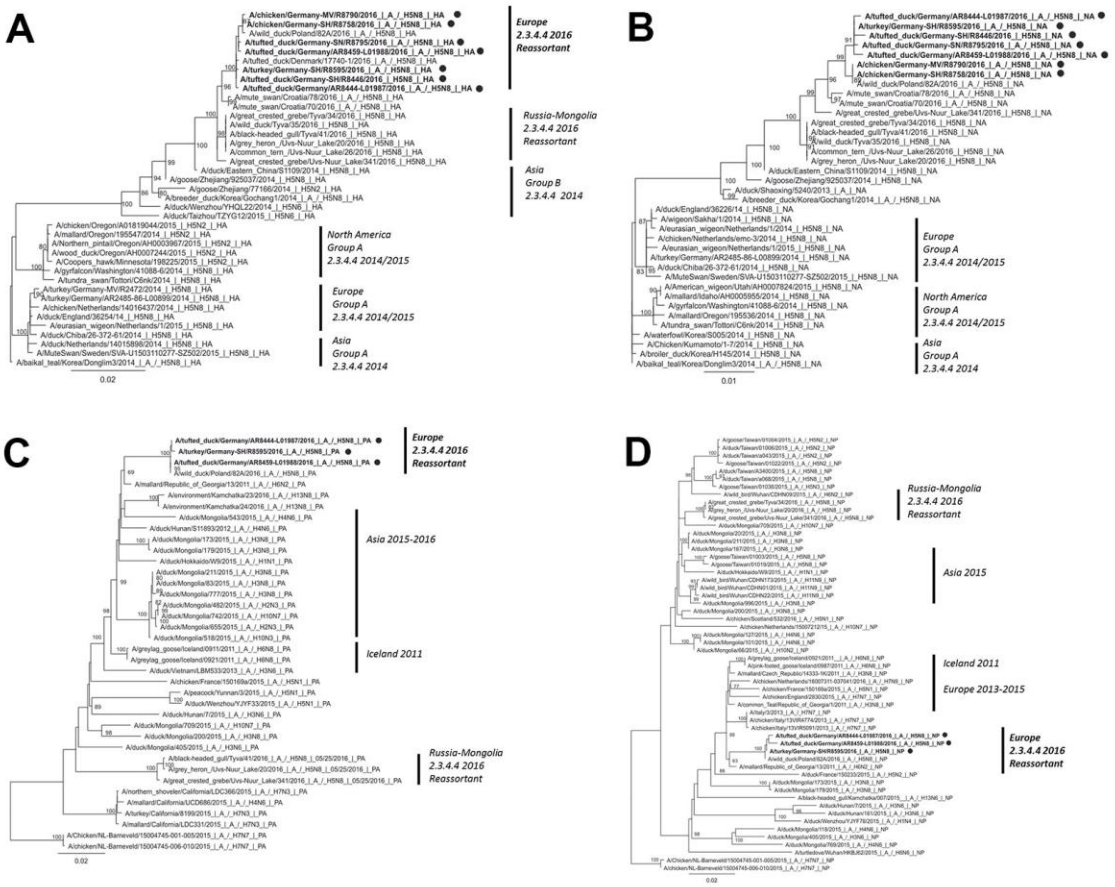

Technical Appendix Figure 2. Phylogenetic analysis of (A) hemagglutin, (B) neuraminidase, (C) polymerase acidic protein and (D) nucleoprotein segment genes done by maximum likelihood trees. Bootstrap values of 1,000 cycles are included. Scale bars indicate nucleotide substitutions per site. Groups are indicated. Viruses that were sequenced in this study are indicated by circles.
